# The complete mitochondrial genome of *Cymothoa indica* has a highly rearranged gene order and clusters at the very base of the Isopoda clade

**DOI:** 10.1371/journal.pone.0203089

**Published:** 2018-09-04

**Authors:** Hong Zou, Ivan Jakovlić, Dong Zhang, Rong Chen, Shahid Mahboob, Khalid Abdullah Al-Ghanim, Fahad Al-Misned, Wen-Xiang Li, Gui-Tang Wang

**Affiliations:** 1 Key Laboratory of Aquaculture Disease Control, Ministry of Agriculture, and State Key Laboratory of Freshwater Ecology and Biotechnology, Institute of Hydrobiology, Chinese Academy of Sciences, Wuhan, P. R. China; 2 Bio-Transduction Lab, Biolake, Wuhan, P. R. China; 3 University of Chinese Academy of Sciences, Beijing, P. R. China; 4 Department of Zoology, College of Science, King Saud University, Riyadh, Saudi Arabia; 5 Department of Zoology, GC University, Faisalabad, Pakistan; University of Parma, ITALY

## Abstract

As a result of great diversity in life histories and a large number of described species, taxonomic and phylogenetic uncertainty permeates the entire crustacean order of Isopoda. Large molecular datasets capable of providing sufficiently high phylogenetic resolution, such as mitochondrial genomes (mitogenomes), are needed to infer their evolutionary history with confidence, but isopod mitogenomes remain remarkably poorly represented in public databases. We sequenced the complete mitogenome of *Cymothoa indica*, a species belonging to a family from which no mitochondrial genome was sequenced yet, Cymothoidae. The mitogenome (circular, 14484 bp, A+T = 63.8%) is highly compact, appears to be missing two tRNA genes (*trnI* and *trnE*), and exhibits a unique gene order with a large number of rearrangements. High compactness and the existence of palindromes indicate that the mechanism behind these rearrangements might be associated with linearization events in its evolutionary history, similar to those proposed for isopods from the *Armadillidium* genus (Oniscidea). Isopods might present an important model system to study the proposed discontinuity in the dynamics of mitochondrial genomic architecture evolution. Phylogenetic analyses (Bayesian Inference and Maximum Likelihood) conducted using nucleotide sequences of all mitochondrial genes resolved Oniscidea and Cymothoida suborders as paraphyletic. *Cymothoa indica* was resolved as a sister group (basal) to all remaining isopods, which challenges the accepted isopod phylogeny, where Cymothoida are the most derived, and Phreatoicidea the most basal isopod group. There is growing evidence that Cymothoida suborder might be split into two evolutionary distant clades, with parasitic species being the most basal split in the Isopoda clade, but a much larger amount of molecular resources carrying a high phylogenetic resolution will be needed to infer the remarkably complex evolutionary history of this group of animals with confidence.

## Introduction

Isopoda is an exceptionally speciose (>10,000) order of crustaceans that mostly but not exclusively inhabit aquatic habitats [1,2]. As a result of great diversity in life histories and a large number of described species, taxonomic and phylogenetic uncertainty permeate the entire order [1,3–7]. As studies relying on morphology and small molecular datasets (such as single gene datasets) did not manage to resolve their phylogeny, this is an indication that phylogenetic resolution provided by the commonly used markers (such as *cox1* or *18S*) is too low to unequivocally resolve the evolutionary history of Isopoda. Therefore, availability of molecular resources carrying a high phylogenetic resolution is indispensable for identification and evolutionary history studies of Isopoda.

Mitochondrial genomes (mitogenomes) carry a large amount of data, which makes them capable of providing much higher resolution than traditionally used morphological and (single-gene) molecular markers, so mitochondrial phylogenomics is increasingly used to address phylogenetic and taxonomic controversies [8–10]. Furthermore, isopod mitogenomes generally exhibit a large number of gene order rearrangements [4,11], and some groups of isopods even possess a non-standard, linearised, mitogenome organisation and unique tRNA-encoding mechanisms [12–16]. As the evolution of mitogenomic architecture appears to be highly discontinuous [17,18], with some major animal taxa exhibiting a highly conserved mitochondrial architecture (most vertebrates being a good example), and other taxa exhibiting a rapidly-evolving architecture [17–21], we hypothesise that isopods might present an important model system to study the complex dynamics of the evolution of mitochondrial genomic architecture. However, isopod mitogenomes remain remarkably poorly represented in the GenBank, with only five complete and 19 partial mitogenomes currently (Apr, 2018) available for the entire order (20 species in total), which presents a major obstacle to their application.

Among the non-represented taxa (taxa from which no mitochondrial genome was sequenced yet) is the entire large (≈366 species and ≈42 genera [22]) family Cymothoidae Leach 1818 (suborder Cymothoida, superfamily Cymothooidea). Species belonging to this family are largely obligate parasites of fishes that feed on host tissues and fluids at least at some stage of their life [1,22,23]. Cymothoid isopods are mostly protandrous hermaphrodites that have a biphasic life cycle: after the free-swimming micropredatory stage, they attach permanently to fishes (or other crustaceans), upon which they change sex and morphology [24]. They exhibit a range of parasitic feeding strategies: on the external body surfaces, in the buccal and opercular cavities, or burrowing into the muscle of their fish hosts [1,22]. The buccal mode sometimes results in the intriguing phenomenon of parasitic castration [25].

Identification and taxonomy of cymothoid isopods are complicated by a number of factors, including morphological similarity, sequential hermaphroditism, sexual dimorphism (females up to three times larger than males), flexible host preference almost completely unrelated to phylogeny, habitat flexibility (sea, brackish and fresh water), global distribution of many species, etc. Along with limited molecular resources currently available, this causes frequent incorrect identifications and misuse of species names, so synonymies and paraphyly are widespread, which is reflected in widely varying estimates of the number of valid species and genera within the family Cymothoidae [1,3,22–24,26–28]. Additionally, although the monophyly of Cymothoida is believed to be supported by morphological data and rejected by molecular data [7], a relatively recent morphological study also failed to find evidence for the monophyly of Cymothoida [29], so it is increasingly likely that the suborder is indeed paraphyletic [5,11,29]. This phylogenetic and taxonomic uncertainty permeates the deep-level phylogeny of Isopoda as well: there are some indications that the position of Cymothoida, which was traditionally regarded as the most derived isopod taxon [4,7,26,30], may be relatively basal within the Isopoda [6,7,27] (throughout the manuscript we use these two terms, basal and derived, to refer to common ancestors, not extant species). The few attempts to apply mitochondrial phylogenomics to study the evolutionary history of Isopoda resolved this suborder relatively ‘centrally’ within the Isopod clade, clustering with Sphaeromatidea and Valvifera [4–6,11]. As only two cymothoid mitogenomes are currently available, both belonging to free-living Cirolanidae species, interpretation and reliability of these findings are severely hampered by the low number of mitogenomes available.

To address this problem, we sequenced and characterised the first complete mitochondrial genome of a parasitic cymothoid isopod, *Cymothoa indica* Schiödte & Meinert 1884 (Cymothoidae), and conducted comparative mitogenomic and phylogenomic analyses. Our results indicate that the availability of this mitogenomic sequence has the potential to advance our understanding of the evolution of mitogenomic architecture and phylogeny of Isopoda, but the amount of available data remains too limited to draw conclusions with confidence.

## Results and discussion

### Genome architecture and characteristics

The complete mitochondrial genome of *C*. *indica* is a circular, 14,484 bp-long molecule, somewhat smaller than most of the remaining complete isopod mitogenomes (Worksheet A in S1 File). Although there is evidence for a linear mitogenomic organisation in *Armadillidium vulgare* [12], an isopod species belonging to a different suborder (Oniscidea), we did not find any indications of such organisation in *C*. *indica* (i.e., all fragments overlapped during the assembly). The mitogenome possesses the standard 13 protein-coding genes and two rRNA genes (*12S* and *16S*), but only 20 tRNA genes, as *trnI* and *trnE* could not be detected (Table 1). A 390 bp-long putative control region (CR) was found between *trnS* and *nad1* genes. The A+T content of the complete mitogenome (63.8%) is average for isopods (54 to 72%; Worksheet A in S1 File). It should be emphasised here again that only five complete mitogenomes were available for the entire order Isopoda when we conducted these analyses, so all comparative analyses in this study were hampered by the fact that the remaining 18 mitogenomes were partial, and should be interpreted with that limitation in mind.

**Table 1:** Organisation of the mitochondrial genome of *Cymothoa indica*.

Gene	From	To	Length	IGR	Start	Stop	Anticodon	Strand
*trnQ*	1	62	62				TTG	-
*trnM*	67	129	63	4			CAT	+
*nad2*	130	1131	1002		ATT	TAG		+
*trnC*	1129	1182	54	-3			GCA	-
*trnY*	1186	1246	61	3			GTA	-
*cox1*	1245	2786	1542	-2	ATG	TAA		+
*cox2*	2830	3508	679	43	ATA	T		+
*trnK*	3509	3569	61				TTT	+
*trnD*	3567	3619	53	-3			GTC	+
*atp8*	3626	3781	156	6	ATA	TAA		+
*atp6*	3775	4450	676	-7	ATG	T––		+
*cox3*	4451	5236	786		ATG	TAA		+
*trnR*	5243	5295	53	6			TCG	+
*trnG*	5296	5353	58				TCC	+
*nad3*	5351	5701	351	-3	ATC	TAA		+
*trnA*	5702	5756	55				TGC	+
*trnV*	5758	5819	62	1			TAC	+
*trnN*	5816	5879	64	-4			GTT	+
*rrnS*	5880	6604	725					+
*CR*	6605	6994	390					
*nad1*	6995	7927	933		ATT	TAA		-
*trnL1*	7937	7996	60	9			TAG	-
*trnL2*	8043	8102	60	46			TAA	-
*trnS*	8100	8158	59	-3			TCT	-
*trnW*	8155	8215	61	-4			TCA	-
*cytb*	8220	9344	1125	4	TTG	TAG		-
*trnT*	9345	9398	54				TGT	-
*nad5*	9398	11095	1698	-1	ATA	TAA		+
*trnF*	11097	11154	58	1			GAA	+
*trnH*	11147	11206	60	-8			GTG	-
*nad4*	11181	12515	1335	-26	ATG	TAA		-
*nad4L*	12514	12781	268	-2	TGG	T		-
*trnP*	12797	12860	64	15			TGG	-
*nad6*	12863	13342	480	2	ATA	TAA		+
*trnS*	13342	13401	60	-1			TGA	+
*rrnL*	13402	14475	1074					-

CR is control region. IGR is intergenic region, where a negative value indicates an overlap.

### Gene order

**Fig 1:**
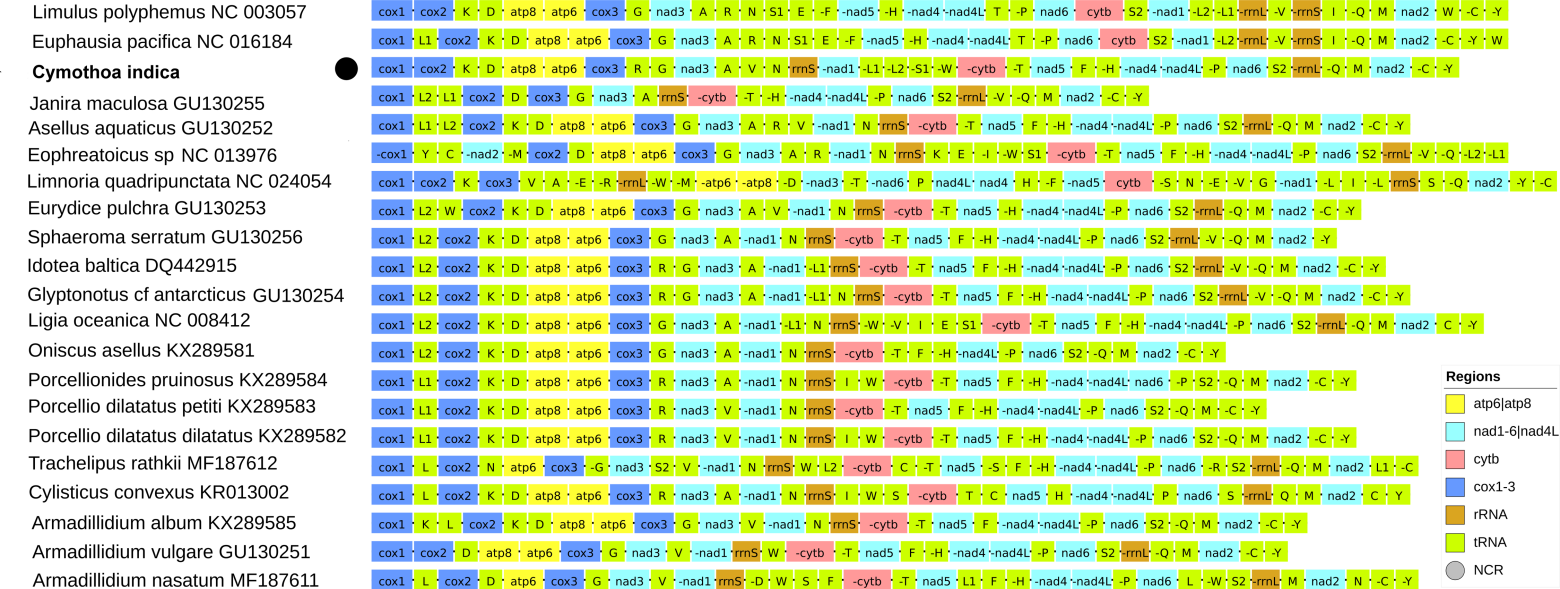
Gene order in isopod mitogenomes. The newly sequenced species, *Cymothoa indica*, is bolded and marked with a black dot. GenBank accession numbers are shown next to the species names, and gene legend is incorporated in the figure. Two outgroups, *Limulus polyphemus* and *Euphausia pacifica*, are also shown.

The ancestral arthropod architecture, almost identical to the one exhibited by *Limulus polyphemus* (Fig 1), has remained almost unchanged for over 400 million years in many crustacean lineages [31], but not among the isopods [32,33]. Gene order rearrangements in this group of animals were discussed in detail in previous studies [4,11,32], so here we only briefly discuss the idiosyncrasies of the new mitogenome. *Cymothoa indica* also exhibits a completely unique order with a large number of rearrangements (Fig 1). The only available isopod mitogenome exhibiting somewhat similar architecture is that of the only other available Cymothoida (family Cirolanidae) species, *Bathynomus* sp. [11]. In terms of PCG and rRNA arrangement, the two mitogenomes are almost identical, with the exception of the position of *nad1*: in *C*. *indica* it is on the minus strand, which corresponds to the putative pancrustacean ground pattern [4,34], but in *Bathynomus* sp. it is on the plus strand (Worksheet B in S1 File). However, the two mitogenomes also differ in the arrangement of a number of tRNAs (L2, A, V, E, W, H, L1, S1), and the location of the putative control region (CR). In terms of uniquely derived gene positions of single species in relation to the putative pancrustacean ancestral gene order discussed by Shen et al. [11], *C*. *indica* shares the unique position of *trnS*-*nad1* with *Bathynomus* sp. In comparison to the putative ancestral isopod architecture [4], *C*. *indica* differs in the arrangement of *nad1* and *12S* rRNA genes, which appear to have switched places, and a number of tRNAs: L2, R, V, S1, W, E and I (the last two are missing). Intriguingly, it shares large, completely conserved (in terms of gene order) segments of the mitogenome of 16/11 genes (*cytb* through *trnY*/*rrnL*) with *Asellus aquaticus*/*Eophreatoicus* sp. respectively, which may bear relevance for resolving the conflicting topologies produced by different datasets for Isopoda (see discussion in the ‘Phylogeny’ section for further details).

Mitogenomic gene order rearrangements are usually attributed to the TDRL (tandem duplication and subsequent evolutionary loss of duplicated genes) mechanism [19,35], but high compactness of isopod mitogenomes indicates that this is not the most parsimonious explanation in this group of animals, as TDRL events result in pseudogenes which are then usually [17,36] ‘erased’ from the mitogenome in the course of evolution [37]. In terrestrial isopods (Oniscidea: *Armadillidium*), a different mechanism was proposed: the atypical organisation of their mitogenomes, which are composed of linear monomers and circular dimers, might facilitate architecture conversions via creation of telomeric hairpins [14,15]. Doublet et al. [14] have characterised the (highly conserved) CR structure in isopod species that undergo (occasional) mitogenome linearization, but the putative CR of *C*. *indica* does not appear to possess any of those features. However, regardless of the different organisation, its CR sequence does contain a large number of palindromes (24 ≥ 6bp), which are required for the formation of hairpins. Therefore, we hypothesise that occasional linearization events in their evolutionary history might be the most parsimonious explanation for the large number of gene rearrangements observed in this species and Isopoda in general.

### tRNAs

Whereas the majority of sequenced metazoan mitogenomes contain the full set of 22 tRNAs, the number of identified tRNA genes often varies in arthropod mitogenomes [16]. Most sequenced isopod mitogenomes appear to possess an incomplete set of tRNA genes [5,32]. As *trnI* and *trnE* appear to be missing from nine and 16 (respectively) of the available mitogenomes, *C*. *indica* is not an outlier in this aspect. Another tRNA gene commonly missing from isopod mitogenomes is *trnW* (13 species), but we annotated this gene upstream of *cytb*, as observed in some other isopods (Fig 1), and successfully folded it into a relatively standard cloverleaf structure. The loss of mitochondrial tRNA genes is usually compensated by the import of nuclear tRNAs [16,38], but some unique features described in oniscidean isopod mitogenomes raise suspicion that these missing tRNAs might be actually encoded in their mitogenomes: heteroplasmic mitochondrial DNA, which may allow for the presence of two tRNA genes with different anticodons at the same locus [15,39,40], and tRNA genes partially or fully overlapping with protein coding genes, that have been reported in the oniscidean *Armadillidium* genus [16]. Although we did find an unusually large overlap (26 bp between *nad4* and *trnH*), we did not find any evidence for the existence of fully overlapping genes, nor for heteroplasmy (by examining electropherograms), so these features appear to remain limited to oniscidean isopods. A 46 bp-long non-coding fragment between *trnL1* and *L2* genes bears high similarity to *trnE* orthologues in other isopods (including the conserved anticodon), but it is missing the 3’ end. We attempted to create a 19-bp overlap with the downstream *trnL2* gene, but the ARWEN tool [41] still did not manage to fold the gene into a cloverleaf structure. Therefore, although the conserved 5’ end and anticodon make us suspect that this tRNA gene might be functional after undergoing post-translational editing [42], as we have no evidence for this, it remains merely a speculation.

### NCRs and overlaps

Mitogenomes of isopods are believed to be very compact, with many overlaps between genes and short non-coding sequences [32]. In agreement with this, we found 12 intergenic regions ranging from 1 to 46 bp in size (excluding the putative control region). The largest was located between *cox1* and *cox2* genes, followed by 15 bp between *nad4L* and *trnP* genes, whereas the remaining nine were smaller than 10 bp (Table 1). Further evidence for this high compactness is a relatively large number (13) of gene overlaps observed, ranging from 1 bp to 26 bp. Eleven of these overlaps, including the largest, between *nad4* and *trnH*, involved at least one tRNA gene. This is expected and believed to be a consequence of lesser evolutionary constraints on tRNA genes [16]. The only overlaps involving two PCGs were those between *atp8*/*atp6* (7 bp) and *nad4*/*nad4L* (2 bp). However, there are several indications that *atp8* and *nad4L* are generally under relaxed evolutionary constraints: both are exceptionally small (in *C*. *indica*: *atp8* = 156bp, and *nad4l* = 268bp); *nad4* and *nad4L* often overlap in mitogenomes of many different groups of animals including isopods [32]; *atp8* is often even completely absent from mitogenomes [19]; and *atp6*/*atp8* overlaps of 4–12 bp were also reported in other isopods [5,11,32]. Therefore, we do not suspect an annotation artefact in either of the two overlaps. In conclusion, this mitogenome can also be characterised as highly compact, which bears relevance for inferring the evolutionary history of its architectural rearrangements.

### Gene features

Most PCGs of *C*. *indica* exhibit sizes and start/stop codons standard for isopods (Worksheet C in S1 File). An exception is *cytb*, which (putatively) uses a non-standard TTG start codon. Alternatively, it might use a standard ATT start codon, but that would create a 12-bp overlap with the neighbouring *trnT* gene. The *atp6* gene exhibited a unique 6 bp-long insertion from positions 56 to 61, but this fragment of the sequence is generally poorly conserved so we don’t deem this as suspicious. Whereas other isopod *atp6* genes end with a TAA stop codon, in the studied mitogenome this codon appears to have mutated into TAT (presuming this is not a sequencing artefact). This is not likely to affect its transcription, as it can still use T—as the stop codon [43]. In *cox1*, a frameshift mutation (or insertion) near the end of the gene appears to have caused a minor extension of the gene: 1542 bp. vs. 1531–1539 bp. in orthologs (*Limnoria quadripunctata* is an outlier with 1596 bp, but as all other genes are highly conserved in size, we suspect an annotation artefact here; Worksheet C in S1 File). Alternatively, it might use a non-standard stop codon, or span only 1524 bp. The 5’ end of *nad5* gene is very divergent, so the start codon is questionable: we selected the standard ATA, creating a 1 bp overlap with the neighbouring tRNA, but if it uses one of alternative start codons, it might start 9, 15, 21, or 24 bp downstream with an ATC.

Several genes exhibit unusually broad size variability in isopods (Worksheet C in S1 File), but in most cases we suspect annotation artefacts, especially as most mitogenomes are incomplete. For example, *cytb* gene exhibits a huge variation in size in the available isopods, from 303 to 1206 bp, but outliers are mostly incomplete, unpublished or unverified mitogenomes, or from a study wherein a large number of mitogenomes were sequenced with the aim to focus on gene rearrangements [4]. An intriguing outlier is *Bathynomus sp*. [11], which exhibited a very divergent 3’ end of the sequence, much longer that the rest of the orthologs. As the authors did not discuss this issue, we also suspect an annotation or a sequencing artefact here. Finally, *nad1* gene also exhibits a suspiciously wide size range (876 to 972 bp). Here again, *Bathynomus sp*. is among the outliers, but in this case its large size (969 bp) is very likely to be an artefact, as it can easily be shortened by 15 bp (to 954 bp) to use the same start codon as most other orthologs, ATA (S2 File). Other three outliers, *Oniscus asellus* (876 bp), *Porcellionides pruinosus* (882 bp), and *Armadillidium album* (972 bp) are unverified and come from the same unpublished study, so their unusual sizes would have to be independently confirmed.

### Phylogeny

**Fig 2:**
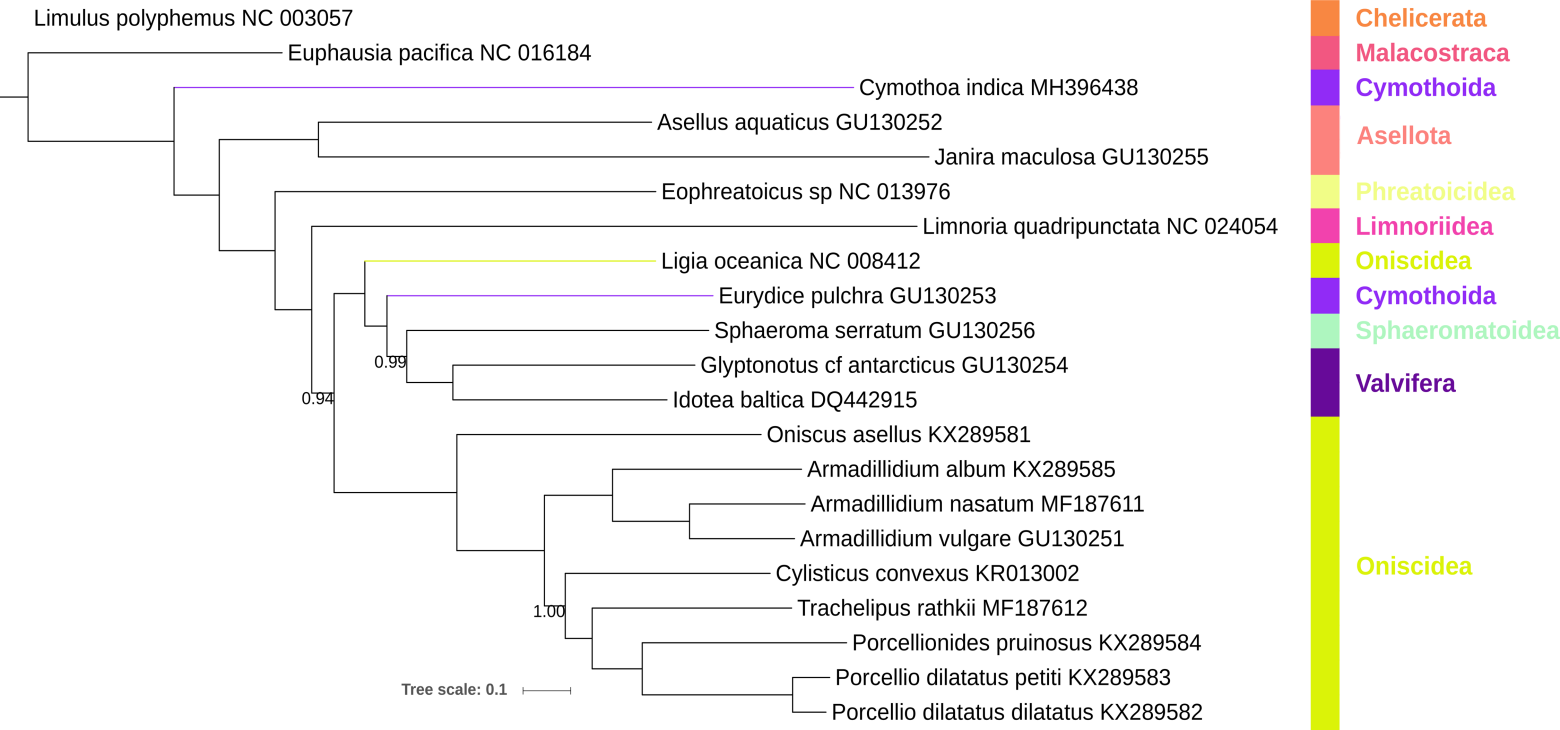
Mitochondrial phylogenomics of Isopoda: Bayesian inference analysis. The analysis was conducted using nucleotide sequences of all genes. *Limulus polyphemus* (branch cropped) and *Euphausia pacifica* are outgroups. Scale bar corresponds to the estimated number of substitutions per site. Bayesian posterior probability values (lower than 1.0) are shown next to corresponding nodes. GenBank accession numbers are shown next to species names. Taxonomic rank (suborder/superfamily) is shown to the right. Coloured branches highlight paraphyly.

**Fig 3:**
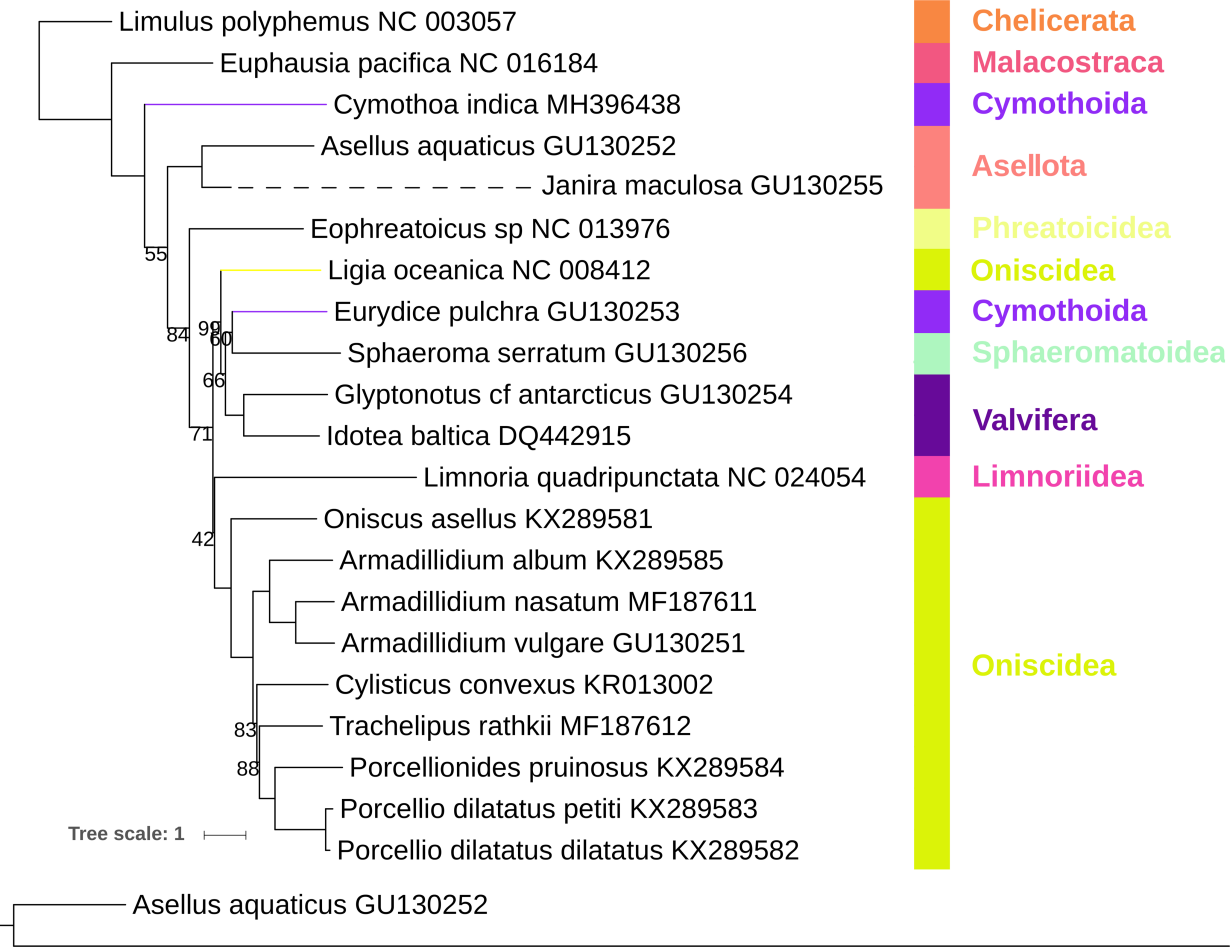
Mitochondrial phylogenomics of Isopoda: Maximum likelihood analysis. The analysis was conducted using nucleotide sequences of all genes. *Limulus polyphemus* and *Euphausia pacifica* are outgroups. Scale bar corresponds to the estimated number of substitutions per site. Bootstrap support values (lower than 100) are shown next to corresponding nodes. GenBank accession numbers are shown next to species names. Taxonomic rank (suborder/superfamily) is shown to the right. Coloured branches highlight paraphyly. *Janira maculosa* branch (dashed line) is shortened, with its original size shown below the phylogram.

The two phylogenetic analyses, Bayesian Inference (BI) and Maximum Likelihood (ML), produced topologies (Figs 2 and 3) differing in three details: the position of *Limnoria quadripunctata* (sister-clade to Oniscidea in ML, a relatively basal position in BI), the relationship of *Eurydice pulchra* and *Sphaeroma serratum* (sister-clades in ML), and the length of the *Janira maculosa* branch (extremely long in ML). In other aspects, the results of the two analyses were congruent, with the BI topology exhibiting very high statistical support, and the ML topology a mix of mostly high and several lower values. The suborder Oniscidea was resolved as paraphyletic by *Ligia oceanica* [32] exhibiting a sister-clade relationship with a cluster of other taxa (*Eurydice pulchra*, Sphaeromatoidea, and Valvifera; see Worksheet A in S1 File for detailed taxonomy). Paraphyly of Oniscidea caused by this genus is a relatively well-established fact [6], and all recent mito-phylogenomic analyses resolved this species approximately in the same position [5,6,11]. Although it is tempting to interpret this as a sign that its position is resolved, we advise that additional mitogenomes belonging to this genus should be sequenced, to exclude the possibility of misidentification or unusual evolutionary rates, before any taxonomic changes are officially proposed. The *Limnoria* genus was described as ‘rogue’ a long time ago [7], and its position also remains unresolved to this day, including by our study. In recent mitochondrial phylogenomic studies it was mostly resolved as a sister-clade to Oniscidea (which corresponds to our ML topology) using different datasets (nucleotides and amino acids) and algorithms (BI and ML) [5,11]. However, Lins et al. [6] resolved it as a sister-clade to all other isopoda (BI, amino acid dataset), and a combined mitonuclear dataset (*18S*, *28S*, and *cox1*) in the same study resolved it as a sister-clade to a derived Asellota clade (Asellota was paraphyletic in that analysis). Therefore, although most mitogenomic analyses resolve it as a sister-clade to Oniscidea, extreme variations in its position indicate that the evolution of this species is very peculiar, and that more molecular data of closely related species should be sequenced to identify the reasons underlying this instability. As the *J*. *maculosa* mitogenome is incomplete (<10,000 bp), we suspected that the long branch produced by the ML analysis may have been an artefact. To test this hypothesis, we removed the four genes (*atp6* and *8*, *nad1* and *5*) missing from *J*. *maculosa* from the entire dataset and re-conducted the ML analysis. This analysis produced a congruent topology (S1 Fig.) and resolved the issue, with the length of the branch comparable to the one produced by the BI analysis.

Most importantly, the newly-sequenced *C*. *indica* species was resolved as a sister group (basal) to all remaining isopods, which challenges the accepted isopod phylogeny, where Phreatoicidea (represented by *Eophreatoicus* sp.) is usually regarded as the most basal split within the Isopoda [4,7,30], and Cymothoida as the most derived group [4,7,26,30]. The most basal position of Phreatoicidea was also challenged by the position of Asellota clade (*A*. *aquaticus* and *J*. *maculosa*), which was derived in relation to Cymothoidae, but basal to Phreatoicidea and all other Isopoda. This topology (Asellota basal to Phreatoicidea) was produced by all other recent mitochondrial phylogenomic analyses as well [5,6,11] (with the exception of an amino acid dataset in [11]). Although all of these topologies support the close relationship of these two clades, and their relatively basal position within the Isopoda clade, none of them show support for the proposed monophyly of a combined asellotan and phreatoicidean clade [4,44]. The suborder Cymothoida was paraphyletic: the other cymothoid species included in the analysis, *Eurydice pulchra* (Cymothoida: Cirolanidae), clustered with Sphaeromatoidea and Valvifera (low support in ML). This position of *E*. *pulchra* is congruent with the topologies produced by most other mito-phylogenomic studies [4–6,11]. A topology highly congruent to ours, including the deep evolutionary split between free-living (Cymothoidae) and parasitic (Cirolanidae) cymothoid species, was produced before using a combined *18S*-morphology dataset [7]. Similar topologies were also produced by two Bayesian analyses of combined mitonuclear gene datasets [6,27], but this was not further discussed by the authors. Intriguingly, that same mitonuclear dataset resolved *E*. *pulchra* within the basal cymothoid clade [6]. The basal position of *C*. *indica* within the isopod clade is further indirectly supported by the similarity in gene order with *A*. *aquaticus* and *Eophreatoicus* sp. (*J*. *maculosa* is incomplete, so it is difficult to assess the level of similarity). Therefore, although the issue cannot be declared resolved yet, it appears that there is increasing evidence from different types of data (gene order, mitochondrial phylogenomics, mitochondrial and nuclear single-marker, and morphological data) for deep paraphyly of the suborder Cymothoida, and for the parasitic Cymothoidae being sister group to all other Isopoda.

## Conclusions

As the absence of a sufficient number of sequenced mitogenomes is currently the foremost limiting factor to their broader application, we sequenced the first mitogenome of a species belonging to the large family Cymothoidae, *Cymothoa indica*. The results of our phylogenetic analyses, which resolved *C*. *indica* as the most basal split in the isopod clade, present a major challenge to the accepted deep phylogeny of Isopoda. There is growing evidence that Cymothoida might be split into two evolutionary distant clades, with parasitic species being the most basal split in the isopod clade. However, the small number of isopod mitogenomes currently available (as well as the minor topological instability observed) prevents us from making any conclusions with confidence. Therefore, a much larger amount of molecular resources carrying a high phylogenetic resolution will be needed to infer the remarkably complex evolutionary history of this group of animals with confidence. Aside from the importance of mitochondrial genomes for taxonomy and phylogenetics, the highly rearranged and unique gene order found in *Cymothoa indica* is a further indication that isopods might present an important model system to study the discontinuous dynamics of the evolution of mitochondrial genomic architecture. Therefore, sequencing of further isopod mitogenomes is strongly urged.

## Materials and methods

### Samples, identification, and DNA extraction

Two adult specimens were collected on 10/07/2017 in Dayawan Town, Guangdong Province, China (22°42’58”– 22°42’56” N; 114°32’16”– 114°32’25” E) from the mouth of a euryhaline fish species, *Mugil cephalus* Linnaeus 1758. Live parasites were kept alive in 0.6% saline as long as possible (one day) to ensure that they were starved, and then stored in 75% ethanol at 4°C. Specimens were morphologically identified under a dissecting microscope as described before [1,3,45,46]. After washing in distilled water, DNA was isolated from one specimen using Aidlab DNA extraction kit (Aidlab Biotechnologies, Beijing, China). This study has been reviewed and approved by the ethics committee of the Institute of Hydrobiology, Chinese Academy of Sciences. As the study involved an unregulated parasitic invertebrate, and as we obtained the samples from already dead fish bought on a local fish market, no permits were required to retrieve and process the samples.

### Genome sequencing and assembly

Ten primer pairs used to amplify and sequence the entire mitogenome were designed to match conserved regions of mitochondrial genes and to overlap by approximately 100 bp (Table 2). Amplification reaction mixture and conditions used were described before [17]; briefly: 50μL with 5 U/μL of TaKaRa LA Taq polymerase (TaKaRa, Japan), 10×LATaq Buffer II, 2.5μM dNTP mixture, 0.2–1.0μM each primer, 60ng DNA template. Conditions: denaturation 98°C/2min, and 40 cycles of 98°C/10s, 50°C/15s, 68°C/1min per kb. When the product was not specific enough, PCR conditions were optimized by increasing the annealing temperature and decreasing the number of cycles. PCR products were sequenced using Sanger method and the same set of primers. All obtained fragments were quality-proofed by visually inspecting the electropherograms and BLASTed [47] to confirm that the amplicon is the actual target sequence. Mitogenome was assembled, annotated, and comparative analyses conducted, roughly as described previously [17,48]. Briefly: assembly was conducted manually using DNAstar v7.1 [49]. In each step we checked whether overlaps were identical, thereby making sure that the mitogenome is circular, and avoiding incorporation of *numt*s [50] into the sequence. The same software was also used to locate the putative ORFs for protein-coding genes. BLAST and BLASTx were used to compare the inferred ORFs with nucleotide and amino acid sequences of available orthologs, and manually determine the exact initiation and termination codon positions accordingly. tRNAs were annotated using tRNAscan [51] and ARWEN [41] tools, and the results checked manually. The annotation was recorded in a Word (Microsoft Office) document, and then parsed and extracted using an in-house MitoTool software [52]. The same software was also used for file conversions (including the creation of the GenBank file) and to generate tables with comparative mitogenomic statistics (including the file used to visualise gene orders in iTOL). Palindromes in the CR were predicted using Palindrome analyser [53]. The mitogenome is available from the GenBank repository under the accession number MH396438.

**Table 2:** Primers used for amplification and sequencing of the mitochondrial genome of *Cymothoa indica*.

Gene/region	Name	Sequence (5’-3’)	Length
*COX1*	LYF1	GCTGGGATATTAGGTCTTAG	1436
	LYR1	GAGTGTTCGGAGGGAGGGAA	
*COX1-COX2*	LYF2	GACGTTATTCAGATTACCCTG	663
	LYR2	GGATAACAAGTTTGTTATCTG	
*COX2*	LYF3	CTGATGAAACTTTTTCATCAC	356
	LYR3	GAAACTATGATTTGCACCAC	
*COX2-COX3*	LYF4	GGACAATCCCATCACTTGGG	1462
	LYR4	TTAGGAGACAATCTTCTATG	
*COX3*	LYF5	GATGTCTCACGAGAAGCAAG	442
	LYR5	GAAAGCCATGAAAACCAGTAG	
*COX3-16S*	LYF6	CAATTATTCTTGGGATTAC	2402
	LYR6	GACCCTAAGAATTTGAAGATC	
*16S*	LYF7	TACGCTGTTATCCCTAGAG	828
	LYR7	CGTACCTTTAGCATTAGGG	
*16S-CYTB*	LYF8	GAAAAGAATTTCACATCTAAAG	5995
	LYR8	CCAAAAGGGTTTCTTGATCC	
*CYTB*	LYF9	GCAATCCCATATATCGGTTC	370
	LYR9	GAAAGTACCATTCAGGTTG	
*CYTB- COX1*	LYF10	CGATCATTTACCCTTATAGAC	2247
	LYR10	CGCCAATTATGATAGGTATAAC	

### Phylogenetic analyses

Three complete and 15 partial isopod sequences were retrieved from the GenBank for the phylogenetic analyses. A basal arthropod, *Limulus polyphemus* [31], and a Malacostraca species basal to Isopoda [32], *Euphausia pacifica* [54], were used as outgroups. To maximize the amount of phylogenetic signal, we conducted the analyses on a dataset containing all genes (PCGs and RNAs). Genes were extracted from GenBank files using MitoTool. Nucleotide sequences of protein-coding genes were aligned in batches (using codon-alignment mode) with MAFFT [55] integrated into another in-house software package—BioSuite [56]. As described before [57], RNAs were aligned by an algorithm that takes secondary structure information into account, Q-INS-i, incorporated into MAFFT-with-extensions software [58]. BioSuite was then used to concatenate the alignments, and another plug-in program in BioSuite, Gblocks [59,60], was used to remove ambiguously aligned regions [59,60]. As a result, although a majority of mitogenomes used for the analysis were incomplete, the final alignment (S3 File) had a relatively low proportion of gaps and undetermined characters (7.68%) and a high number of distinct alignment patterns (9,641). Best partitioning scheme and evolutionary models for partitions (GTR+I+G, GTR+G; S4 File) were selected using PartitionFinder [61], also implemented in BioSuite. Phylogenetic analyses were conducted using Bayesian Inference method implemented in MrBayes 3.2.6 [62] (default settings, two parallel runs, five million MCMC generations) and Maximum Likelihood method implemented in RAxML 8.1.21 [63] (1000 rapid bootstrap replicates). Phylograms and gene orders were visualized in iTOL [64]. WoRMS database [65] was used as the authority for the taxonomic nomenclature.

## Supporting information

S1 File Comparative analyses of isopod mitogenomes. Worksheet A: taxonomy and basic statistics for all available isopod mitogenomes. Worksheet B: ancestral gene orders. Worksheet C: gene statistics for all available isopod mitogenomes (+ outgroups): gene sizes, start and terminal codons. Species are represented by acronyms of their binomial scientific names (see footnote). The new sequence (*C*. *indica*) and outgroups are shaded grey.
(XLSX)

S2 File Alignment of selected isopod *nad1* orthologs. (FAS)

S3 File Alignment of isopod mitogenomes used for phylogenetic analyses. (FAS)

S4 File PartitionFinder results: Partitioning and model selection. (TXT)

S1 Fig Maximum likelihood phylogenetic analysis conducted on a partial dataset. Four genes missing from *Janira maculosa* were removed from the entire dataset: *atp6*, *atp8*, *nad1* and *nad5*. See caption for Fig 3 for other details.
(TIF)
