# Supplementary figures and images for: The complete mitochondrial genome of Cymothoa indica has a highly rearranged gene order and clusters at the very base of the Isopoda clade
Source: PLoS One. 2018 Sep 4;13(9):e0203089. doi: 10.1371/journal.pone.0203089 (PMC6122833; doi:10.1371/journal.pone.0203089)

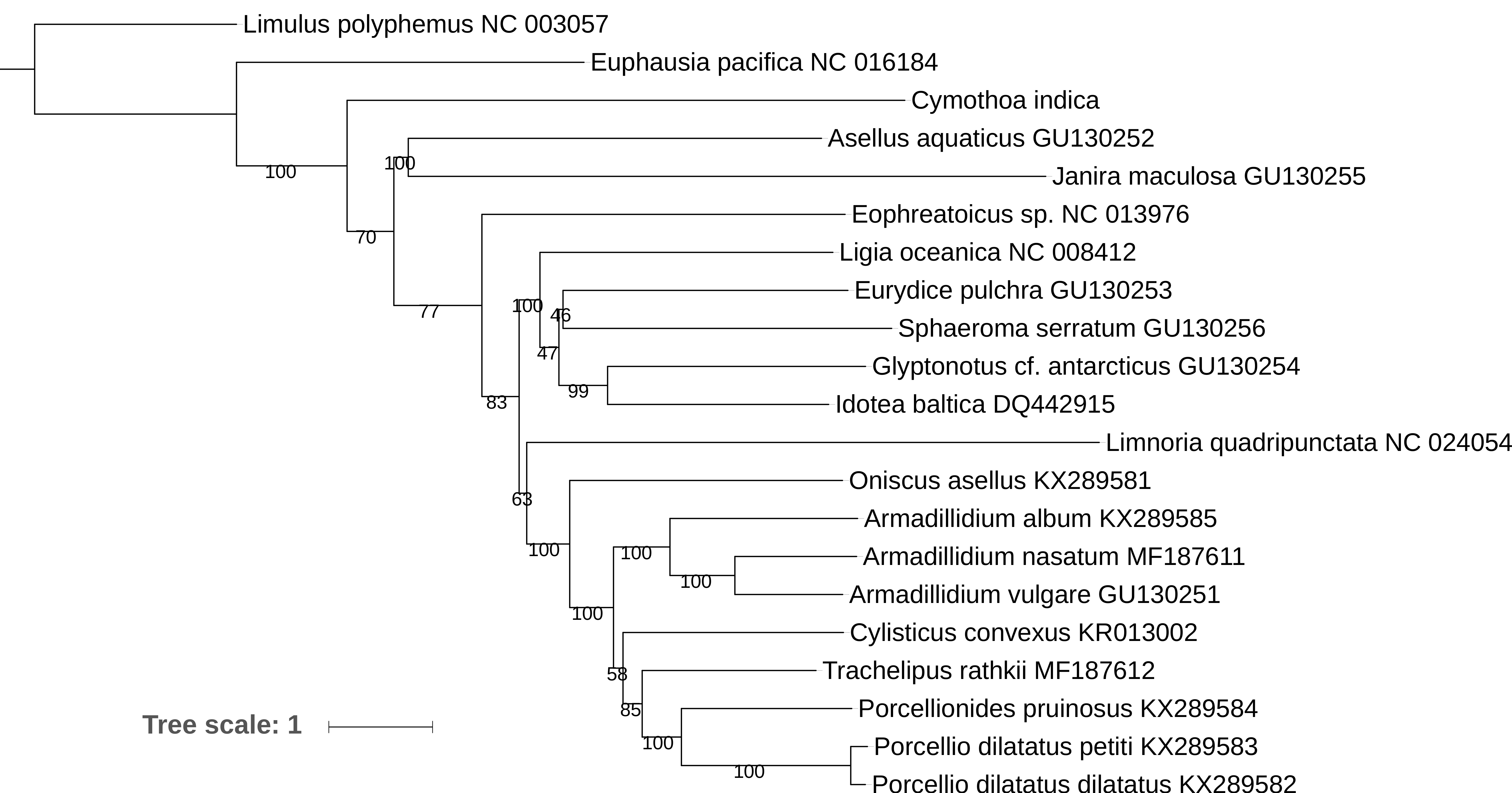

Supplement: S1 Fig — Four genes missing from Janira maculosa were removed from the entire dataset: atp6, atp8, nad1 and nad5. See caption for Fig 3 for other details. (TIF) [file pone.0203089.s005.tif]
